# Daily requirement of softgel thyroxine is independent from gastric juice pH

**DOI:** 10.3389/fendo.2022.1002583

**Published:** 2022-09-26

**Authors:** Camilla Virili, Silvia Capriello, Ilaria Stramazzo, Nunzia Brusca, Maria Giulia Santaguida, Lucilla Gargano, Maria Flavia Bagaglini, Giovanni Bruno, Carola Severi, Marco Centanni

**Affiliations:** ^1^ Department of Medico-surgical Sciences and Biotechnologies, ‘‘Sapienza’’ University of Rome, Latina, Italy; ^2^ Endocrine Unit, Azienda Unità Sanitaria Locale (AUSL) Latina, Latina, Italy; ^3^ Department of Translational and Precision Medicine, Gastroenterology Unit, ‘‘Sapienza’’ University of Rome, Rome, Italy

**Keywords:** Hypothyroidism, levothyroxine softgel, levothyroxine malabsorption, gastric juice pH, atrophic gastritis

## Abstract

**Background:**

Softgel levothyroxine (LT4) preparation showed a better *in vitro* dissolution profile at increasing pH as compared to tablet LT4 preparation. Clinical studies suggested a better performance of softgel LT4 preparation in patients with gastric disorders but whether this finding is related to gastric juice pH variation *in vivo* is not known.

**Methods:**

Twenty-eight hypothyroid patients (24F/4M; median age=50 treated with tablet LT4 (median dose= 1.65 µg/kg/day) and with stable thyroid stimulating hormone (TSH) values on target (<0.8-2.5> mU/l) have been shifted to softgel LT4 preparation. The dose of softgel LT4 has been titrated to obtain a similar individual serum TSH value. All subjects followed a specific treatment schedule, taking LT4 in fasting condition and then abstaining from eating or drinking for at least 1 hour. Owing to the presence of long-lasting dyspepsia or of already known gastric disorders, all patients underwent endoscopy, upon informed consent. Gastric juice has been collected during endoscopy to measure gastric pH. Then we plotted the dose of LT4 with the gastric pH obtained *in vivo*, before and after the switch tablet/softgel preparation in all patients.

**Results:**

Upon the switch tablet/softgel preparation, the therapeutic LT4 dose was very slightly reduced (-6%) in the whole sample. However, the individual variations revealed the existence of two populations, one without any dose reduction (A) and the other showing a dose reduction >20% (B). Upon matching with the actual gastric pH, patients with normal pH (A: n=17; 14F/3M, median 1.52) no showed a lower softgel LT4 requirement. Instead, among patients with reduced gastric acid production (B: n=11; 10F/1M, median pH 5.02) the vast majority (10/11; 91%, p<0.0001) benefited from a lower dose of softgel LT4 (median = -23%, p<0.0001). Interestingly, the dose of LT4 in tablet correlated with pH value (Spearman’s ρ =0.6409; p = 0.0002) while softgel dose was independent from gastric juice pH (Spearman’s ρ =1.952; p = 0.3194).

**Conclusions:**

These findings provide evidence that softgel LT4 preparation is independent from the actual gastric pH in humans and may represent a significant therapeutic option in patients with increased LT4 requirement, owed to disorders impairing the gastric acidic output.

## Introduction

Levothyroxine in tablet formulation is one of the most prescribed drug in the world (1), shows a narrow therapeutic index and is often a lifelong treatment. This implies a careful individualization of the dose, which should take into account not only the anthropometric features but the mode of ingestion, the physicochemical characteristics of LT4 preparation and the interfering agents or disease, as well (2–5). The key of thyroxine treatment efficacy is the absorption process (6) which in turn depends on the gastrointestinal status of patient (gastric acidity, motility, drugs and nutritional interferences, comorbidity etc.) (7–9). At gastric level, the absorption of LT4 is negligible but disaggregation and dissolution of the tablet occur in the stomach (10–12), thus influencing the amount of the hormone available to be absorbed at the intestinal level (7). In particular, evidence has been provided that gastric acid secretion may interfere with tablet LT4 efficacy both *in vitro* and *in vivo* (13–18). In fact, despite the active ingredient is the same in all pharmaceutical preparations of the hormone, the different vehicle and/or excipients of some preparations (3, 19) may affect the processes leading to the active ingredient liberation. Novel preparations have been made available from the pharmaceutical industries in the attempt to optimize LT4 absorption (3). Among these, the softgel preparation showed an *in vitro* dissolution profile that seemed influenced by medium pH variations to a lesser extent as compared to two different tablet formulations, (13). A camera observed the dissolution process of this formulation during endoscopy in a young healthy volunteer: 10 minutes following ingestion, the volume of the capsule was reduced by 50% and 21 minutes after intake, the softgel preparation totally disappeared (20). From a clinical standpoint, this formulation performed well in subjects with gastroparesis (21), in patients showing a high LT4 requirement due to proton pump inhibitors (PPIs) use (22) and in patients with increased need related to different patterns of gastritis (23). In this latter study, we demonstrated a decreased need for thyroxine in 2/3 of enrolled patients. In the remaining third, the requirement of tablet and softgel formulation was substantially the same and we suggested the usefulness of direct measurement of the gastric juice pH (23). Subsequently, evidence has been provided *in vivo*, that gastric juice pH is one of the major determinants of the minimal effective dose of tablet LT4 (8). Therefore, we decided to assess the relationship between softgel LT4 requirement and the actual gastric juice pH in oral thyroxine treated patients.

## Patients and methods

### Patients: Inclusion and exclusion criteria

Patient selection was carried out through a careful evaluation of the clinical and follow-up data of each of the subjects treated in our tertiary Endocrinology Unit.

Inclusion criteria: a) hypothyroidism due to Hashimoto’s thyroiditis; b) Body mass index <30 kg/m^2^; 3) long term treatment with LT4 in tablet; 4) TSH target ​​in the range 0.8-2.5 mUI/l and stable for at least 2 years; 5) concurrent presence of gastric symptoms and/or dyspepsia.

Exclusion criteria were the presence of: a) goiter, thyroid cancer, previous thyroidectomy; b) intestinal diseases or bariatric surgery; c) severe comorbidities or use of drugs able to interfere with the metabolism of LT4; d) pregnancy or lactation. The diagnosis of Hashimoto’s thyroiditis was based on the presence of at least two out of three of the following criteria: the presence of high anti-thyroperoxidase antibodies (anti-TPOAb) titers (>200 U/ml) and/or characteristic ultrasonographic pattern and/or hypothyroidism.

### Design of the study

The study group was a follow up of a previously published study (8) in which we correlated the actual gastric juice pH of 61 patients to their tablet LT4 requirement of a single brand. As described in (8), these were hypothyroid patients with Hashimoto’s thyroiditis in need for thyroxine treatment. These 61 patients underwent upper endoscopy with multiple biopsies and gastric juice pH evaluation, as suggested by the gastroenterologist, because of long lasting dyspepsia or suspicion of gastritis. Following these procedures, 28 of these patients (24 females/4 males) (median age=50, IQR=43-65; median weight = 68 Kg, IQR= 60-76 Kg) accepted the switch from the LT4 tablet formulation to the softgel formulation and represented our study group. All these subjects followed a specific treatment schedule, taking the tablet in fasting condition and then abstaining from eating or drinking anything other than water for at least 1 hour, as previously described (24). The LT4 dose was determined as the one that allows achieving a target TSH in the range 0.5-3.0 mU/l. L-thyroxine requirement was tailored according to age and weight starting from 1.0 mcg/Kg/die. Patient’s compliance with this treatment schedule was verified using a questionnaire and by interview at each visit.

## Methods

Levels of serum free thyroxine were detected by commercial assay (Thermo Scientific, BRAHMS FT4 RIA, Hennigsdorf, Germany). Serum TSH levels were assayed by commercial assay (Thermo Scientific, BRAHMS TSH RIA, Hennigsdorf, Germany) (normal range, 0.4–4.0 mU/l). Serum anti-TPOAbs were measured by commercial assay (Thermo Scientific, BRAHMS anti-TPOAbs, Hennigsdorf, Germany) (normal range: <60 U/ml).

### Upper Endoscopy

All patients underwent upper endoscopy in the morning, as described in (8), fasting from at least eight hours. Briefly, all subjects were sedate using intravenous midazolam (range to 2-5 mg). Seven biopsies were collected in the stomach, two from antrum (smaller and greater curve), one from the *incisura angularis*, two from midbody (smaller and greater curve) and two from fundus during retroversion (25, 26). To exclude duodenal pathologies such as celiac disease, two biopsies were obtained from the second duodenal portion. The evaluation of gastric alterations was based on the updated Sydney scoring system (27) evaluating the acute inflammation (neutrophil infiltrate), chronic inflammation (mononuclear cell infiltrate), glandular atrophy, intestinal metaplasia, Helicobacter pylori infection (27). The protocol was consistent with the principles of the Declaration of Helsinki and the study has been performed within the usual diagnostic workup of each single patient, upon written informed consent and according to the local ethical rules. The study was approved by the Sapienza University Ethical Committee. STROBE guidelines were followed to describe this observational study.

### Gastric juice aspiration and gastric pH value assessment

This procedure has been performed as previously described (8). Five ml of gastric juice were immediately aspirated in fundus and mid-body (greater curve) by means of a sterile Teflon catheter and collected into a sterile trap connected with the suction line of the endoscope. The pH of gastric juice was measured with a glass electrode pH meter and afterwards titrated with a 1N solution of NaOH to evaluate H^+^ concentration in each sample.

### Statistical analysis

Statistical significance was defined as a two-sided p-value <0.05 for all analyses which were carried out by using the INSTAT Graphpad TM 3.06 software (Graphpad Inc., San Diego, CA) for Windows.

Descriptive analysis was based on median and interquartile range (IQR) values for continue variables and on relative frequencies used for qualitative variables. Statistical differences have been calculated by Paired Student’s *t* test to compare different variables.

Fisher’s exact test has been used to compare proportions of different groups. Spearman’s ρ test has been used to assess correlation between variables.

## Results

**Figure 1:**
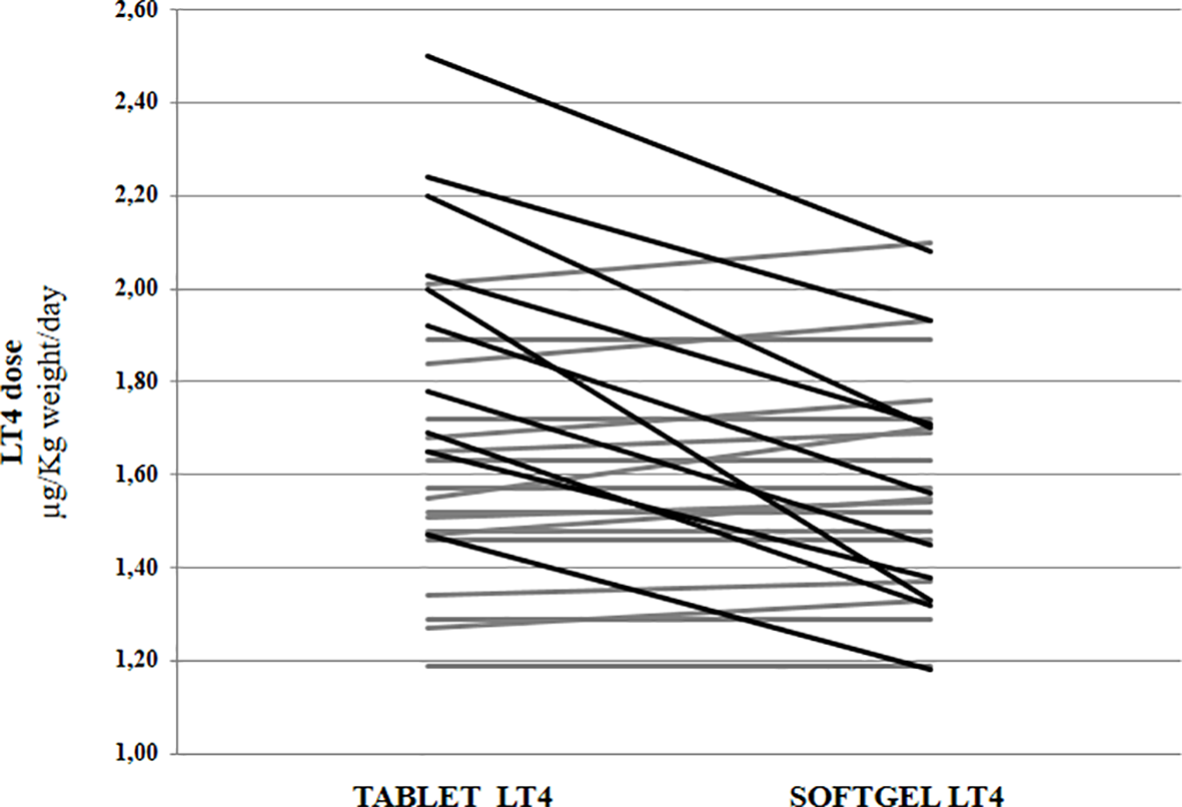
Levothyroxine daily requirement in patients treated with tablet preparation and following the switch to softgel formulation. Black lines indicate patients with a reduction of requirement higher than 20%. In grey are indicated patients without a significant requirement variation.

**Figure 2:**
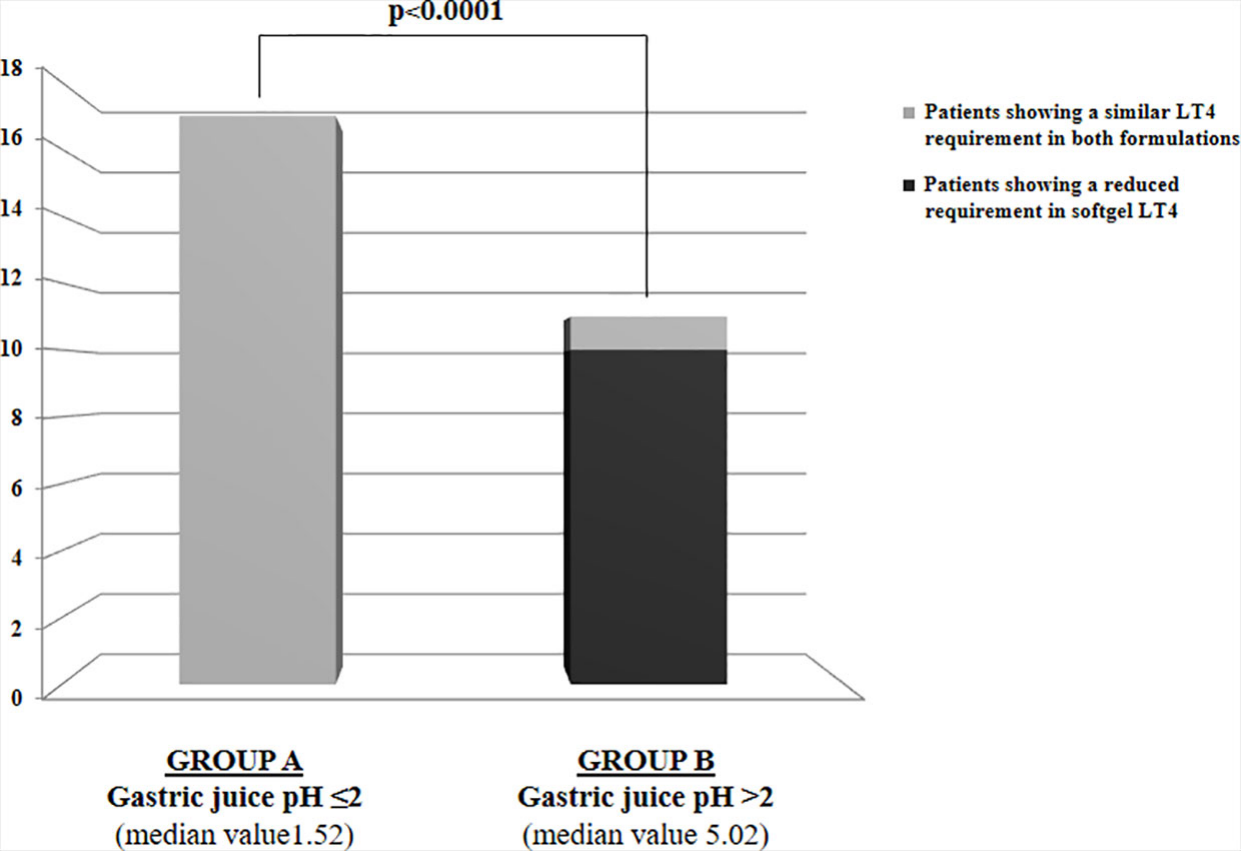
LT4 requirement variations following the switch to softgel formulation in the whole sample of patients subdivided in two groups based on gastric juice pH: Group A with gastric juice pH ≤2 and Group B with gastric juice pH>2.

**Figure 3:**
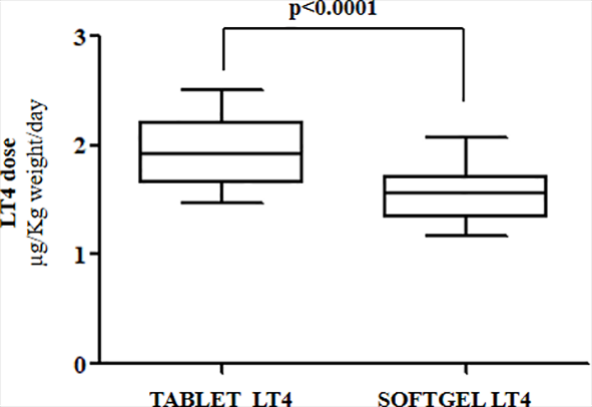
Median LT4 requirement in patients with gastric juice pH>2 (Group B) before and after the switch to softgel formulation.

Euthyroidism was achieved in all patients in tablet LT4 (median TSH=1.3 mU/l). Subsequently, all patients switched from tablet to softgel LT4 formulation and serum TSH was measured again after 6 to 8 weeks. Then, dose adjustments, if needed, were made according to the serum TSH values obtained. To reach a similar serum TSH (1.30 vs. 1.89 mU/l; paired t test p=0.6899) the LT4 requirement was very slightly lower in softgel than in tablet LT4-treated patients (1.56 vs. 1.65 μg/kg/day). This difference was statistically significant (paired t test: p=0.0106), but the percentage of reduction was only of 6%, lower than expected from previous pharmacokinetic and clinical studies (14, 23). This perplexing result prompted us to analyze the dose trend in each single patient: after the switch, we observed a LT4 requirement reduction of more than 20% in 10 out of 28 patients, while the remaining 18 patients did not change their LT4 requirement, as depicted in **Figure 1**. To explain the reason of this clear-cut double population, according to previous evidence (8, 23), we plotted the dose of LT4 with the gastric pH obtained *in vivo* through endoscopy. In the whole sample the median pH was 2.01 (IQR =1.38-3.54) being the average of H^+^ concentration of 52 mEq/l considered normal in fasting human beings (28, 29). However, a wide range of pH values has been observed in the whole sample: from a minimum value of 1.09 (114 mEq/l H^+^) up to a maximum value of 7.45 (zero mEq/l H^+^). Being gastric pH a major determinant of oral LT4 dose (8), we subdivided the whole sample in two groups based on the actual gastric juice pH and then the dose of LT4 has been re-analyzed. We used a cut-off of pH 2.0 to define two different groups of patients, according to (28, 29). Groups consisted in: A) (gastric pH<2) 17 patients (14 females, 3 males) with a median pH of 1.52 (IQR: 1.28-1.70) (median H^+^ =mEq 78) and B) (gastric pH>2) 11 patients (10 females, 1 males) with an average 5.02 (IQR: 3.13-6.72) (median H^+^ =mEq 21). No difference were observed about age, sex, weight and functional thyroid data (not shown). Subsequently, we examined the relationship between the decreased requirement of softgel LT4 and the actual gastric juice pH. The dose of LT4 was unchanged in all 17 patients with normal pH and significantly decreased in 10 out of 11 patients with high gastric pH (Fisher’s exact test: p<0.0001) (**Figure 2**). So far, upon the switch between tablet and softgel preparation, the LT4 dose appeared to be reduced by 23% in the group B (paired t test: p<0.0001) (**Figure 3**).

**Figure 4:**
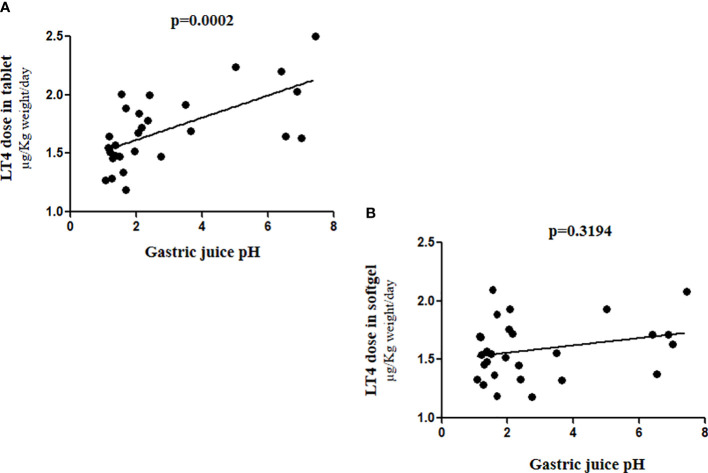
Correlation between gastric juice pH values and LT4 requirement in **(A)** tablet and **(B)** softgel formulation.

The correlation curve helped us to explain the different efficacy of these two different LT4 formulations. The slope of the curve confirmed, as already described (8), a statistically significant correlation between the gastric pH and the LT4 requirement in tablet formulation (Spearman’s ρ=0.6409 p= 0.0002), whereas the softgel LT4 formulation appears to be independent from that variable (Spearman’s ρ =0.1952 p= 0.3194) (**Figure 4**).

## Discussion

The main finding of the present study comes from the evidence that the softgel LT4 preparation is less sensitive to pH values above the normal fasting ones. Overall, these data provides a pathophysiologic explanation to the lower requirement of softgel LT4 observed in patients with impaired gastric acidic output.

The possible impact of gastric juice pH on LT4 bioavailability is actually linked to the effect on disaggregation and dissolution of the pharmaceutical form, which are both sensitive to pH variations (12). However, the role of the different excipients in determining the effectiveness of different formulations should not be disregarded. Indeed, the effectiveness of an oral dosage form depends upon the intrinsic ability of the drug to dissolve in the fluids of the gastrointestinal tract, prior to being absorbed into the circulation (12). Therefore, the rate of dissolution is pivotal to this process independently from the different LT4 formulations. In the softgel LT4 preparation the active ingredient is dissolved in glycerol surrounded by a gelatin envelope (3). However, *in vitro* studies also demonstrated that the pH of the medium in which thyroxine is solubilized, is able to influence both the LT4 ionization and conformational status, determined by the relative orientation between the phenolic and the tyrosyl rings (30, 31). The ionization status of the molecule leads also to the possible creation of aggregates affecting its solution behavior (31). To note, thyroxine molecule is an amphipathic compound, with a hydrophilic and a lipophilic part (7, 32). It features three ionizable groups with different pK_a_ and the variations of medium pH lead to the appearance of different molecular micro-species characterized by different solubility (7, 32). Even the crystalline conformation of the molecule is affected by the different medium pH, as polymorphs are characterized by different degrees of solubility (30). Up to now, whether these variations are able to affect the intestinal LT4 absorption is not known: the transporters responsible for LT4 uptake by enterocytes are, in fact, not fully characterized (8) but it is conceivable that the LT4 ionization status might affect the efficiency of that process. Furthermore, gastric hypochlorhydria may influence the pH of small intestine content, leading to intestinal bacterial proliferation (33), affecting in turn thyroid hormones enterohepatic recycling (34).

From a pharmacokinetic point of view, the softgel formulation of LT4 is bioequivalent to the tablet one in healthy subjects (35) but not necessarily in patients with altered gastric juice pH (14). In fact, some clinical studies evaluated the major effectiveness of this LT4 formulation in patients treated with proton pump inhibitors, with gastroparesis or with different forms of gastritis (21–23). Hypochlorhydria is more frequent than previously reputed (36–38). In fact, in half of the world population, H. Pylori infection may occur at least once in a lifetime, often associated with an impairment of gastric acid secretion (36). A further example comes from the frequent co-ingestion of L-T4 and PPIs. These are two of the most frequently prescribed drugs worldwide and in a study involving more than five thousands LT4 users, PPIs were co-prescribed in about 70% of them (39, 40). The impairment of acid secretion is usually stable in patients with chronic atrophic gastritis (GCA), chronic pangastritis or gastritis of the body-fundus (25) which is often associated with Hashimoto’s thyroiditis (16, 26, 38) and with malabsorption of other nutrients including iron (41, 42).

The different degrees of gastric acid secretion impairment in the above conditions help to explain the different efficacy of softgel as compared to tablet formulation in these patients.

The limitations of our study stem from the small number of patients as well as from the lack of follow up, owing the invasive procedures requested to measure the gastric pH *in vivo.*

The strength of the present study comes from:

- the direct evidence that softgel LT4 preparation is independent from the actual gastric pH in humans.
- the reliable use of this formulation in patients with LT4 refractoriness owed to altered gastric acid secretion
- the reduction of dose adjustments needs, obtained by using this softgel formulation, which may lead to decreased healthcare expenses (43).

Based on these results, softgel LT4 formulation may represent a first line treatment in some categories of patients in need for a better absorption profiles and more rapid and/or stabile achievement of therapeutic target due to disorders impairing the gastric acidic output.

## Data availability statement

The raw data supporting the conclusions of this article will be made available by the authors, without undue reservation.

## Ethics statement

The studies involving human participants were reviewed and approved by Comitato Etico dell’Università Sapienza di Roma Prot. 0262/2021. The patients/participants provided their written informed consent to participate in this study.

## Author contributions

CV, MC, and SC conceived, designed the study, and wrote the final version of the manuscript. GB performed endoscopies, GB and CS collected gastroenterological data and revised the manuscript. MS, LG, and SC organized the data and wrote the first draft of the article. MB, NB, and IS performed the literature search and contribute to the analysis of data. All authors contributed significantly to the article and approved the submitted version.

## Conflict of interest

MC was invited lecturer in international symposia and received honorarium from IBSA, Pambio Noranco, CH.

The remaining authors declare that the research was conducted in the absence of any commercial or financial relationships that could be construed as a potential conflict of interest.

## Publisher’s note

All claims expressed in this article are solely those of the authors and do not necessarily represent those of their affiliated organizations, or those of the publisher, the editors and the reviewers. Any product that may be evaluated in this article, or claim that may be made by its manufacturer, is not guaranteed or endorsed by the publisher.
